# Supplementary material for: The Construction and Exploration of a Comprehensive MicroRNA Centered Regulatory Network in Foxtail Millet (Setaria italica L.)
Source: Front Plant Sci. 2022 May 6;13:848474. doi: 10.3389/fpls.2022.848474 (PMC9121102; doi:10.3389/fpls.2022.848474)
Supplement: Supplementary file 4 [file Data_Sheet_4.zip › Sit-miR3979_Seita.6G056400.1_752_TPlot.pdf]

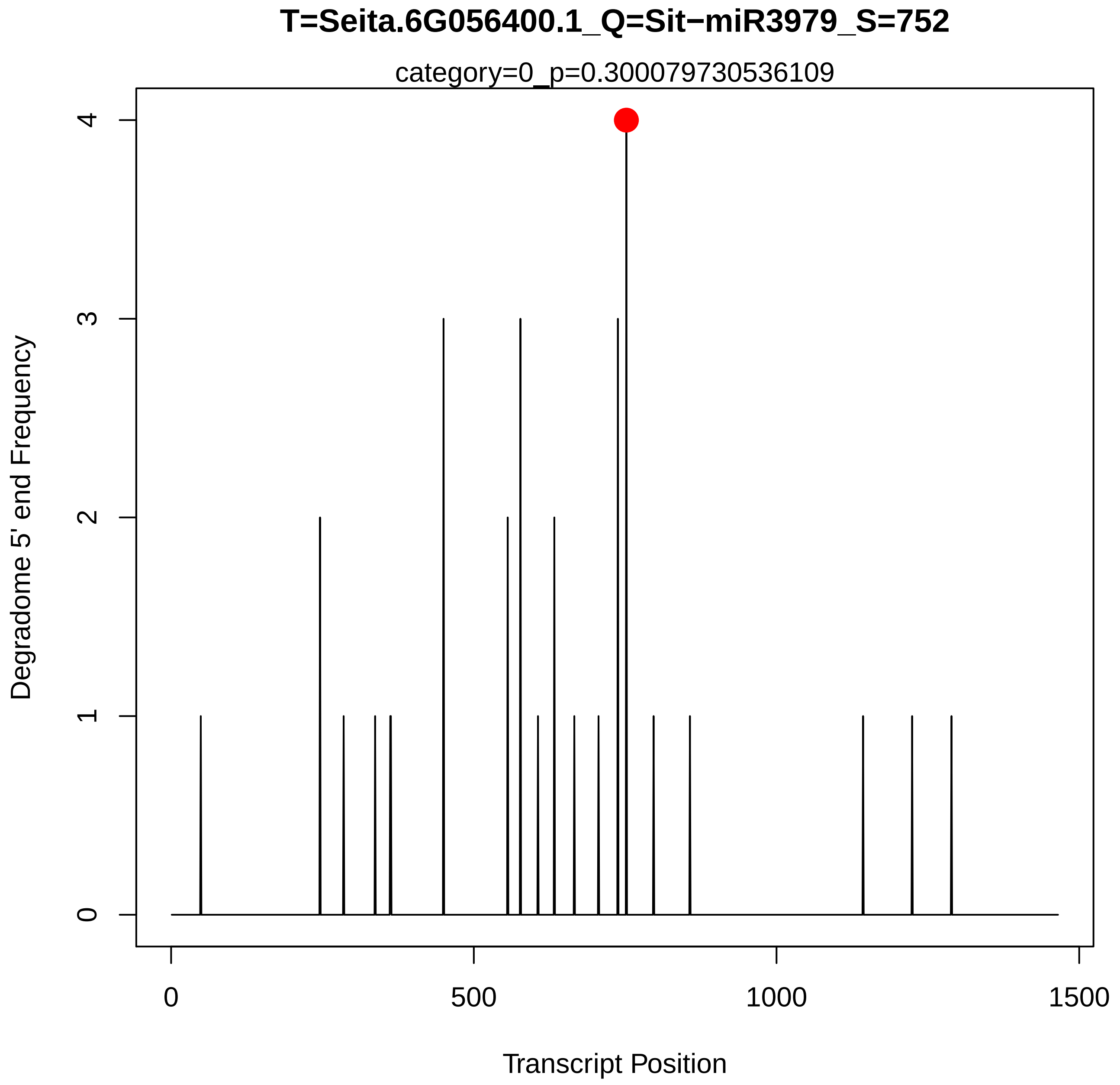

T=Seita.6G056400.1_Q=Sit−miR3979_S=752
category=0_p=0.300079730536109
Degradome 5' end Frequency
0
1
2
3
4
0
500
1000
1500
Transcript Position
